# Clinical Reports of the Surgical Practice of the Glasgow Royal Infirmary

**Published:** 1833-01-01

**Authors:** 


### XIII.

### Clinical Reports of the Surgical Practice of the Glasgow Royal Infirmary. By *John Macfarlane,* M.D. Senior Surgeon to the Royal Infirmary, &c. &c. &c.

This valuable work arrived at too late a period to admit of an extended notice in the last number of this Journal. We did, however, draw attention to it in our Periscope department, and in the present number we shall avail ourselves freely of the practical facts which it contains. We have already expressed the satisfaction which we feel, and which we are sure all who wish well to their profession must feel too, at the publication of works of this description. They are hospital reports in a most authentic form, and they offer a collection of facts which enable us to ascertain with considerable certainty the actual state of medicine or surgery. We have previously dwelt upon this subject, and the benefits accruing to the profession from the statement of a man's experience in the straight-forward manner displayed by Dr. Macfarlane, must be sufficiently obvious without any laboured exposition on our part. We pass, therefore, to the consideration of the cases related by our able and industrious author. We shall select what appear to us to be most interesting, and present them in as condensed a form as possible.

Let us turn to the affections of the urinary and genital apparatus. And first of affections of the bladder.

### I. Calculus in the Bladder.

"Without entering into a critical examination of the various plans which have been at different times adopted, for the removal of urinary concretions by surgical operation, I shall briefly state the mode of procedure adopted in the following cases:—A large curved staff, having a wide and deep groove on its convex side, was passed into the bladder, the patient was secured in the usual way, and an assistant appointed to fix the pelvis, by pressing against the iliac bones. A long narrow double-edged scalpel was then thrust into the perinæum, between the erector penis and accelerator urinæ, and its point carried steadily forward till it rested in the groove of the staff. The external incision was completed, by carrying the knife downwards and outwards between the tuberosity of the ischium and the anus, care being taken to depress the handle, and to elevate the point of the scalpel, so as to withdraw it from its deep position on passing the rectum. By this incision, the staff was always so fairly and completely exposed, that, on introducing the finger into the upper part of the wound, farther dissection was seldom required; the same scalpel was, therefore, run along the groove into the bladder, and the preliminary steps of the operation finished in a few seconds, by dividing the membranous part of the urethra and prostate gland in the usual direction.

The extraction of the stone is often the most difficult, and generally the most uncertain part of the operation; and on the care and caution with which this is performed, will the safety of the patient not unfrequently depend. When the calculus is of the usual size, when it is encysted, or grasped by spasmodic contraction of the bladder, the difficulties are greatly increased. The first three cases illustrate these points." 105.

It seems that Dr. Macfarlane's method of operating differs from that in general use in its first steps. He does not dissect for the groove of the staff, but makes a stab in the perinæum with a sort of catlin, completing the external incision, we presume, by cutting outwards, and passing the same *unbeaked* instrument into the bladder. Let this be recollected in perusing the cases.

Case 1. *Large Stone successfully extracted.* "J. T., æt. sixty, admitted 27th July, 1826. Had experienced pain and difficulty in voiding urine for more than five years. On examination, this was found to depend on the presence of a calculus. It was ascertained to be of a large size, in consequence of the dull, obtuse sound produced by striking it with a metallic instrument, the large space it occupied in the bladder, and its apparent weight and extent when examined per anum. Doubts were entertained as to the possibility of extracting it by the lateral operation. This was, however, ultimately decided on, as from the duration of the disease, and the consequently contracted and thickened state of the bladder, the high operation seemed impracticable.

On the 30th, the lateral operation was performed, and after a little difficulty, an oval-shaped calculus was extracted. It measured six and three-fourths inches in the transverse, and eight inches in the longitudinal circumference, and weighed six ounces and five drachms. The wound healed slowly, and he was dismissed on the 23d of October.

Besides the difficulty experienced in the extraction of so large a stone, it was found, that from rheumatic stiffness of the knees and hip-joints, it was impossible to separate the thighs above a few inches from each other, or to bend them upon the abdomen, so as to obtain the usual space for the external incisions." 106.

CASE 2. *Stone thought to be encysted—Extraction—Cure.* J. R. æt. 23, admitted Sept. 28th, 1827. He had laboured under symptoms of stone for 20 years, the symptoms having seldom been severe, but occasionally aggravated by violent exertion or intemperance. The calculus was only discovered on passing the forceps deeply and raising the point; it was rough, irregular, and felt only and constantly on the right side of the fundus. The instrument could be moved around what seemed a projecting portion of stone, and from one irregular point to another. Varying the position—repeated sounding—injection of the bladder produced no change in the situation of the calculus, and Dr. M. conceived that it was encysted. The lateral operation was determined on, and it was expected that the projecting part of the stone might be laid hold of, and that so the whole might be extracted.

"I operated on the 6th of October. After the prostate was divided, the stone could not be discovered by the finger; but, on introducing a pair of long forceps, and pushing them up to the right side of the fundus, as far as the handles would permit, it was firmly grasped, and brought down with ease to the edge of the divided prostate, evidently bringing the fundus of the bladder along with it, when the instrument slipped. This occurrence happened frequently, and the forceps had always to be introduced in the same direction, and to the same depth as at first; showing that the stone had not changed its position, and adding to the belief of its being encysted. This opinion was still farther confirmed, by retaining the calculus as near the wound as possible, by the forceps held in the left hand, while the right fore-finger was introduced by the side of the blades, and the calculus found to be firmly grasped by the bladder. The finger nail could be introduced between the edge of the cyst and the stone, round its whole circumference. To have employed much force in attempting to tear away the calculus, would have been dangerous,—the bladder might have been inverted, and separated from its neighbouring connexions. I therefore selected a pair of forceps with thin blades, introduced them to the necessary depth, carrying the handles well back towards the sacrum, laid hold of the stone, and, by a slow wriggling motion, moved them along its surface, with the view of insinuating their points between the border of the cyst and the stone. When this appeared to be accomplished, the handles were gradually separated in several directions, so as to produce dilatation, if possible, without lacerating the parts, and the stone was again grasped, and, with a little force, at length extracted. It was of the mulberry kind, and weighed one and a half ounce. The one side was smooth, whilst the opposite was nodulated; and, although there was no distinct neck, there was a deep groove traversing nearly two-thirds of its circumference, exactly where it was grasped by the bladder. But little blood was lost, and the patient bore the operation with great fortitude. An elastic tube was introduced through the wound into the bladder, and retained for thirty hours, after which the urine passed by the wound for three weeks, when it closed. He had no bad symptoms, and, about the middle of November, was again at his employment." 108.

We think it most probable that the stone in this instance was really encysted. However, the case is interesting because the difficulties attending its management are displayed. Our readers are no doubt aware that, when contraction of the bladder prevents the easy removal of the stone, it has been recommended to leave the stone in the bladder until the spasmodic action of the latter shall have ceased. Amongst others, Mr. Lizars has published cases in which this method was successful. Dr. Macfarlane gives a case of this description.

Case 3. *Operation à deux temps—Cure.* A. P. æt. 17½, Nov. 1831. He had suffered severely from calculus for two years, and voided large quantities of the triple phosphate. The lateral operation was performed on the 25th, and the stone found fixed above the pubes on the right side.

"Having failed (says Dr. M.) to dislodge it by one or two cautious attempts, and not being able to seize it with the forceps, I ordered the patient to bed, trusting that, in a few hours, the contraction of the bladder would cease, and the stone drop into a depending part, and be afterwards extracted. In five hours after the operation, I was hurriedly sent for, and found that his urine was completely obstructed. On removing a coagulum from the wound, and attempting to pass the finger into the bladder, I found a calculus, about the size of a walnut, sticking in the wound of the prostate, which was easily extracted by the forceps. In ten days, he had a severe attack of cynanche tonsillaris, which ended in suppuration; but the febrile excitement gradually abated, and the wound filled up, although slowly." 109.

In the three succeeding cases there was enlarged prostate gland, a complication not in itself dangerous. We shall notice them very briefly.

Case 4. The patient was 63, and had laboured under stone-symptoms for three years. The prostate gland was as large as half an orange, smooth and soft. The calculi, six in number, and each as large as a small marble, were lodged behind the prostate, and, to remove them, it was necessary first to push them back into the bladder. An elastic tube was retained in the bladder for 24 hours after the operation. The patient was cured in a month.

Case 5. W. W. æt. 64, Nov. 12, 1831. The symptoms had existed for six years, were urgent, attended with fever, and repeated attacks of hæmorrhage from the bladder. The prostate, especially the left lobe, was enlarged, indurated, irregular.

"After a few days' preparatory treatment, the lateral operation was performed on the 18th, and a rough, oblong calculus extracted. It measured two and a fourth inches in length, one and a half inches in breadth, and weighed one ounce and one drachm. The transverse artery of the perinæum bled freely, and there was rather profuse venous hæmorrhage on dividing the prostate, amounting in all to about eight ounces. This continued after he was put to bed, and produced a feeble pulse and a bleached countenance. An elastic tube was introduced, and the wound filled with sponge; but there still continued a good deal of oozing, and he had repeated threatenings of syncope for twelve hours. On the morning of the 19th, the sponge accidentally slipped from the wound, and the bleeding returned; it was therefore re-introduced, and retained till the evening of that day, after which time there was no farther hemorrhage. He had considerable febrile excitement for several days, and nearly a fortnight elapsed before the wound began to granulate; it, however, completely closed, and he was dismissed in excellent health." 111

Case 6. The patient was æt. 16—the symptoms had existed for six years, and were urgent—pain in the left kidney following the ureter—urine loaded with mucus and mortar-like deposit—the prostate hard, painful, and twice its natural size. After proper remedial measures the operation was performed. The stone was rough, soft, weighing nearly one ounce and a half. An elastic tube was retained for thirty hours. The patient was dismissed cured at the end of January.

"I think favourably of the practice of introducing a tube into the bladder, and retaining it till the tract of the wound be consolidated. We shall thus obviate the risk of urinary extravasation, prevent premature cohesion of the sides of the wound, or closure of it by clots of blood, and facilitate the regular discharge of the urine. In the last case, but for the use of a tube, the escape of urine would have been prevented, the wound having been closed by the great protrusion of fat which took place in the course of the external incision. This practice will be found to prevent the wound, both in the bladder and externally, from adhesion by the first intention, an occurrence which I have met with in two cases. It is not, however, desirable, nor indeed is it safe, to favour this premature closure of the wound; by which means the passage of the urine along the penis becomes for some days both painful and difficult, giving rise to local irritation and constitutional excitement, as well as to the danger of extravasation. When the tube has not been introduced, and the urine for some hours after the operation appears to be discharged with difficulty, I do not hesitate to pass the finger through the wound into the bladder, and thus to destroy any adhesions that may exist. I have seen three cases, in which, by a neglect of this simple procedure, extravasation of urine into the cellular texture of the pelvis was produced, giving rise to fatal peritonitis. One of these, which occurred to myself, I shall now detail." 112.

Case 6. *Urinary Extravasation fatal.* G. G. æt. 18, admitted Dec. 11th, 1826. The symptoms had existed for two years, and were unusually severe, with extreme irritability of the bladder. After soothing treatment for some days the operation was performed on the 17th, and a very rough mulberry calculus, weighing 65 grains, extracted.

In the evening he had pain in the wound, uneasiness in the hypogastrium, and desire to void urine. The adhesions were separated by the finger, and about four ounces of urine discharged. In the night he again had difficulty, but next morning the water flowed freely. He had then rapid, indistinct pulse—anxious countenance—swelling above the pubes without pain on pressure; he had had a slight rigor. The finger was passed into the bladder, but no coagulum found. *Castor oil.* In the evening, increased pain above pubes and in wound; in the left inguinal region a small spot where pressure was intolerable. Leeches—*Cal. and rhub.—bath.* At 1, a. m, was bled to 14ozs, syncope, blood not being buffed or cupped. Lower half of abdomen swollen and painful on pressure—pulse 140—brown fur on tongue. *Leeches—cal. and ant.—purgative salts.* At. 7, p. m. swelling of belly somewhat diminished—pulse scarcely to be counted. *Blister.* Next day, said he was better;—face hippocratic—pulse intermittent—belly tympanitic. Died at midnight, 83 hours after the operation.

"*Dissection.* Bowels were distended with air, and there existed a few patches of inflammation on the peritoneal covering of the ileon. There were about four ounces of sero-purulent fluid effused into the pelvis; but the traces of peritoneal inflammation were here very slight and obscure. The cellular texture, surrounding the bladder, especially on its anterior surface, and that covering the left psoas muscle, was broken down, and loaded with pus and urine. The bladder was thickened, and its mucous coat highly inflamed, ecchymosed, and, in some places, ulcerated." 114.

The case is a good example of rapidly fatal inflammation of the cellular tissue, from extravasation of urine. Dr. Macfarlane makes two remarks that perfectly coincide with those previously made by Mr. Brodie in his published lectures—first, that depletion is frequently carried to an injurious extent after lithotomy—and secondly, that peritonitis is not such a common sequence of the operation as many suppose it to be. There can be little or no doubt that diffuse inflammation of the cellular tissue has been and continues to be often mistaken for peritonitis. We must not imagine that this cellular inflammation is always produced by extravasation of urine. Patients occasionally die of it, children more especially, and yet no satisfactory evidence of effusion of urine can be met with after death. We know that after injuries and after operations, diffuse cellular inflammation of other parts occurs. The anatomical characters and the general symptoms are similar, and it is not absurd to suppose that the mere operation of lithotomy may give rise to diffuse cellular inflammation, independently of any extravasation of urine. The cellular texture of the pelvis, we must recollect, is apt to be much contused and injured by the introduction of the forceps and removal of the stone. We trust that, by drawing attention again and again to the opinions of Mr. Brodie on these subjects, we shall rouse many surgeons from the supine belief in the frequency of peritonitis after lithotomy, into which they would really appear to have fallen.

Case 7. *Lithotomy fatal in consequence of Osseous Tumor in the Mesentery.* R. C., æt. 67, admitted Jan. 23d, 1827. He had the symptoms of stone with more than ordinary severity. He had, on several occasions, voided small calcareous particles, about the size of a pin's head, and one hard smooth yellow stone, of the size and shape of a kidney-bean. A large sound was introduced, and from the rattling in the bladder there seemed to be several stones. The urine deposited a small quantity of flaky sediment. The prostate was much enlarged. The bowels were obstinately costive, the abdomen somewhat tympanitic. The symptoms had existed about nineteen months. After purgation and some other treatment, the operation was performed. Six entire and three broken calculi were extracted, the largest being oval and about the size of a walnut. The prostate felt almost like cartilage, and was so elastic as to close the wound in the bladder, like a valve. Three arteries bled freely, especially the transversalis perinæi. It was too deep to be tied, but the bleeding was arrested by pressure. An elastic tube was introduced into the bladder, the body kept cool, the thighs separated. Nothing particular had occurred by that evening, but next morning he was affected at intervals with severe spasmodic pains in the abdomen. In the evening he had pain on pressure above the pubes, with accelerated pulse. *Leeches and enema.* 31st. Continued to complain of fixed pain above pubes, with violent expulsive efforts. *Tube withdrawn—castor-oil—leeches.* On Feb. 1st had six stools, but symptoms continued. A large elastic tube was passed into rectum to let out the air, without success. On 2d, had a rigor, followed by fever and nausea. *Calom. and opium—V.S.—fomentations.* On next day was better, but on the 5th the flatulence and tenesmus were increased—he had had several scybalous stools, with excruciating pain during their evacuation. Complained of feeling a large hard body in upper part of rectum. From this time till 10th there was little change—his countenance was pale and exhausted. The treatment consisted in frequent and copious injections. On the 15th he had a violent attack of pain referred to the rectum, but nothing could be felt there. He was ordered hyosciamus and camphor, and other soothing measures. For three days he seemed better, but on the morning of the 22d he was found dead in his bed.

" *Dissection.* On opening the abdomen, a hard tumour was discovered lying over the last lumbar vertebra, between the laminæ of the mesentery, near the inferior part of the ileon, and which pressed on the sigmoid flexure of the colon, where it is about to become rectum. The surrounding mesentery exhibited no thickened or diseased appearance, and only adhered to the surface of the tumour by loose cellular attachments, easily destroyed by the finger. It was about the size of a small lemon, of a hard bony feel and appearance, and a very irregular shape. When sawn through, the exterior part was evidently bone, and varied in thickness, at different parts, from a quarter to half an inch, whilst the centre was filled by a yellowish-white substance, in appearance and consistence like adipocire, intersected in various directions by spiculæ of bone. Two small cavities in the centre were lined with innumerable transparent, needle-like crystals, which, however, disappeared after the tumour was dried, and before I had an opportunity of submitting them to chemical analysis. The mucous coat of the bladder was considerably thickened, of a dark vascular-plaited appearance, especially about the neck, and coated by a muco-purulent secretion. There was a tumour at the fundus about the size of a small marble, containing purulent matter, which issued into the cavity of the bladder, through two fistulous openings in the mucous coat at that part. The prostate gland was enlarged, and firmer in texture than natural, but without the fibrous appearance of scirrhus. The mucous coat of the rectum was highly inflamed, and there was considerable induration and thickening of parts between this gut and the base of the bladder.

This dissection afforded a satisfactory explanation of what had been previously only matter of speculation. The long-continued and painful tenesmus was obviously to be referred to the pressure of the osseous tumour, on the commencement of the rectum, producing an impediment to the regular discharge of the feces, tympanitic swelling of the abdomen, and great irritation. From the situation and connexions of this tumour, it would appear, that when the diaphragm and abdominal muscles were called into action in expelling the feces, it would be forced back on the termination of the colon, by the pressure of the surrounding parts, and not only impede the feculent evacuations, but also, from its extreme hardness and inequality, irritate and injure the bowel in no small degree.

In scrofulous habits, the mesenteric glands are sometimes filled with calcarious matter; but bony depositions are stated by Dr. Baillie, (Morbid Anatomy, p. 134,) to be of rare occurrence. The few recorded cases of this disorganization, which I have had an opportunity of examining, appear to have originated in disease of the glands of the mesentery, and to have been complicated with organic disease of the bowels. Dr. Donald Monro narrates a case in the Medical Transactions, (vol. ii. p. 361,) in which all the mesenteric glands, varying in size from a pea to a walnut, were hardened and ossified. They were not, however, as in the case above detailed, made up of one large firm osseous tumour, but, 'like spongy carious bones, they were composed of a number of small pieces, joined together by membranes.'" 120.

Professor Thompson, analyzed the stone and the bone. The former was chiefly uric acid. The latter was not adipocire, nor muscle, nor ligament, but "a little fat was separated," "and, when heated, it behaved like cartilage." We are afraid this does not add much to our stock of positive knowledge.

We relate all the fatal cases, because such are always instructive. Yet they are not so much so as they might be. Dr. Macfarlane, for instance, makes no mention of the state of the wound in the bladder, its size, direction, &c. Yet this is important; for surgeons have attached great importance to the prostatic incision. Mr. Brodie believes that if the left half of the prostate be wholly cut, or, at all events, if the prostatic capsule be cut, the risk of cellular inflammation is very much augmented. Other surgeons have said that the prostate should rather be extensively cut than stretched. How are such points to be decided, if they should ever admit of a decision? By a rigid observation of facts, and by that only. But if facts are superficially observed or recorded, they might almost as well be withheld. We address this remark to the profession, rather than to Dr. Macfarlane individually.

Case 8. *Lithotomy—Extensive Disease of the Prostate and Bladder—Death Eight Weeks after the Operation.* J. N. æt. 68, admitted Jan. 18th, 1832. Has very frequent desire to make water, always accompanied by prolapsus of the rectum, which he must replace before the catheter can be introduced, and by paroxysms of excruciating pain extending along the penis to the glans—urine containing a copious whitish-coloured sediment, and occasionally small coagula, decidedly alkaline, loaded with muco-purulent secretion, and depositing mortar-like masses. On introducing a sound, a calculus detected. Prostate about the size of half an orange, hard, irregular. A large catheter passing easily, the prostate was thought to cover the vesical orifice of the urethra, like a valve, which was the fact. The general health was impaired. He had suffered from the complaint for several years, and for the last three and a half required the introduction of the catheter nearly every second hour.

"After the use of acids, frequent doses of the Oleum Ricini, the hip-bath, anodyne enemata, &c., by which the bowels were unloaded, and the appearance of the urine greatly improved, I was reluctantly induced, by the earnest entreaties of the patient, who was suffering most acutely from the disease, and by the recommendation of a consultation, to try the chance of an operation. This was accordingly performed on the 5th of February, and a rough calculus, about the size of a walnut, extracted. He bore the operation, which lasted about a minute, with remarkable firmness, and not more than six ounces of blood were lost. Before he was removed from the table, a large gum elastic tube was introduced through the wound into the bladder. He continued to improve steadily from the third day after the operation. The elastic tube was withdrawn every fourth day, when its extremity was usually coated with calcarious matter, and its cavity filled with viscid mucus. It was near the end of February before I could pass a catheter along the penis into the bladder. This was also removed every third or fourth day, cleaned and re-introduced,—it being thought more likely to hasten the closure of the wound, by retaining the catheter in the bladder for several days at a time, than by introducing it every two or three hours, when the urine required to be drawn off. On the 25th of March, the wound was nearly closed; he had no pain; his bowels were kept regular by medicine; his appetite was good; he had improved decidedly in flesh and strength; and his general health was better than it had been for many years. He was, in fact, considered to be out of all danger from the operation; and the symptoms of diseased bladder were much less troublesome than could have been expected. On the following day, (the 26th,) he complained of slight pain in the anus, which became prolapsed. On the 28th, as the urine was turbid, and contained a thick chalky sediment, the catheter was withdrawn, and introduced only when he felt inclined to empty his bladder. There was some febrile excitement; the pulse was about a hundred; the tongue dry and furred, and the bowels loose. He complained of pain and confusion of head; and his eyes were suffused. These symptoms increased; vomiting, hiccup, delirium, and subsultus tendinum supervened. His tongue and teeth were covered with sordes; and, altogether, his appearance resembled that of a person labouring under typhus gravior. He died comatose, at the hour of visit, on the 3d of April,—eight weeks and two days having elapsed from the time of the operation.

The body was inspected on the 4th, and the following morbid appearances discovered. The bladder was greatly thickened, contracted, and indurated; its mucous coat was covered with a dark-coloured muco-purulent secretion, and in one or two places it was slightly ulcerated: the rugæ were in some places so deep, as to produce the appearance of small sacculi. All the lobes of the prostate gland were enlarged, and of a hard, almost cartilaginous texture: the middle one, which projected into the bladder, was of a pyriform shape, and completely covered the orifice of the urethra: on its apex there was a small patch of superficial ulceration. The right kidney contained a cyst, the size of a pigeon's egg, which was filled with a straw-coloured fluid, like urine. It was seated in the cortical substance of the gland, but did not communicate with its pelvis. The inferior half of this kidney was soft and disorganized. The left kidney was small, and its natural structure completely changed. It contained a number of hard, greyish-coloured tubercles, as also pus and small calcareous particles. The rectum, immediately within the sphincter, was surrounded by a large indurated ring of hemorrhoidal tumours of a deep purple colour." 124.

It will be observed, that in the preceding case the prostate was ulcerated, the bladder in a state of chronic inflammation, and the kidney affected with "greyish-coloured tubercles, pus, and calcareous particles." Mr. Brodie has warned surgeons against operating on patients with ulcerated prostate, and relates, if we remember right, three cases in which the operation proved fatal. In this instance the ulceration was probably not so extensive. In patients who have suffered long from stricture, or stone in the bladder, it is not an uncommon thing to find one or both kidneys studded with numerous small abscesses. They appear to commence as small, solid, yellowish depositions, which gradually soften, and become as they enlarge converted into pus, or pus takes the place of the more consistent deposition. The kidney thus affected would seem to be in a state of chronic inflammation, its capsule peeling off with facility, and its substance being more or less injected. Patients thus affected bear operations on the urinary organs extremely ill. We have seen a man die from the introduction of a catheter, and after death this condition of kidney was found, with a small abscess at the neck of the bladder, opening into the urethra. This patient had an old and bad stricture of the urethra. Such persons die with typhoid symptoms, and, as in other affections of the kidney, there is a great disposition to coma.

Dr. Macfarlane adds to the cases already mentioned, four in which lithotomy was successfully performed in children. The fourth is the only one possessed of interest. The child in that instance was in a very unfavourable state of health, and every thing seemed to conspire against the success of an operation. But it was successful and completely so. We regret that we cannot find room for the details, but we insert with pleasure the following exposé of Dr. Macfarlane's sentiments on an important question in practice.

"It is hardly possible to meet with a case more unfavourable for operation than the one now detailed. I believe that all those surgeons who are ambitious to acquire and maintain a reputation as successful lithotomists, and who are careful in selecting their patients, would have declined operating on this case, as well as on some of the others already narrated. I am not satisfied, however, that any surgeon, from a morbid anxiety about his own reputation, and a wish to be able to exhibit a long list of successful cures, is justifiable in denying to the diseased, even in doubtful and unfavourable cases, that professional assistance which both humanity and science claim at his hands. I am by no means an advocate for the knife, unless there is a prospect of its being successfully employed; nevertheless, when it is the only means we possess of prolonging existence,—why, even when the prospect is not inviting, should any selfish feelings prevent us from having recourse to it?" 128.

The succeeding case is one of much interest, and our readers will readily see why.

Here the present notice of this work must terminate, but many of the remaining cases will be noticed in the Periscope department of this Journal at convenient opportunities. None have been more earnest than ourselves in recommending our provincial surgeons to publish the results of their experience, and we feel convinced that, if they follow the example now set them by Mr. Fletcher of Gloucester, Mr. Clement of Shrewsbury, and Dr. Macfarlane of Glasgow, they will materially contribute to the advancement of their profession, and raise the reputation of provincial surgeons and of themselves. It is not every one who can write a book worth reading on any one subject; but all well-informed and candid practitioners can communicate what they have seen, and contribute to the accumulating stores of facts. The advice which we should give would be this:—Be scrupulously accurate—be concise without niggardly and unprofitable brevity—and endeavour to group and arrange your cases so methodically, that while each individual shall have its value as a separate fact, they shall still act en masse, and serve for those generalizations without which science can never be materially advanced. Thus we would have surgeons give the general expression of their experience with remedies or with operations, whilst they select such particular cases for particular notice as they deem most fitting. In fine, we hope, ere long, to witness a systematic plan of clinical reporting adopted by our provincial brethren. None will be more happy than ourselves to aid this most excellent object in every possible manner.

